# Persistent Organic Pollutants and Type 2 Diabetes: A Critical Review of Review Articles

**DOI:** 10.3389/fendo.2018.00712

**Published:** 2018-11-27

**Authors:** Yu-Mi Lee, David R. Jacobs Jr., Duk-Hee Lee

**Affiliations:** ^1^Department of Preventive Medicine, School of Medicine, Kyungpook National University, Daegu, South Korea; ^2^Division of Epidemiology and Community Health, School of Public Health, University of Minnesota, Minneapolis, MN, United States; ^3^BK21 Plus KNU Biomedical Convergence Program, Department of Biomedical Science, Kyungpook National University, Daegu South Korea

**Keywords:** chemical mixtures, diabetes, insulin resistance, obesity, organochlorine pesticides, persistent organic pollutants, polychlorinated biphenyls

## Abstract

Low dose persistent organic pollutants (POPs) have emerged as a new risk for type 2 diabetes (T2D). Despite substantial evidence from human and experimental studies, there are several critical issues which have not been properly addressed by POPs researchers. First, as POPs exist as mixtures, findings about POPs from human studies should be interpreted from the viewpoint of lipophilic chemical mixtures which include both measured and unmeasured POPs. Second, as POPs can directly reduce insulin secretion of beta cells, the role of POPs may be more prominent in the development of beta-cell dysfunction-dominant T2D rather than insulin resistance-dominant T2D. Third, there are multidimensional interrelationships between POPs and adipose tissue. Even though POPs are now considered as a new risk factor for T2D, independent of obesity, POPs and obesity are mechanistically linked to each other. POPs are involved in key mechanisms linking obesity and T2D, such as chronic inflammation of adipose tissue and lipotoxicity with ectopic fat accumulation. Also, POPs can explain puzzling human findings which suggest benefits of obesity because healthy adipose tissue can be protective by reducing the amount of POPs reaching other organs. Fourth, non-linear dose-response relationships between POPs and T2D are biologically possible. Although POPs are well-known endocrine disrupting chemicals (EDCs), mitochondrial dysfunction may be a more plausible mechanism due to unpredictability of EDC mixtures. As adipose tissue plays a role as an internal exposure source of POPs, how to manage POPs inside us may be essential to protect against harms of POPs.

## Introduction

Persistent organic pollutants (POPs) include a wide range of organic compounds which are resistant to degradation by chemical or biological processes. As a result, they are highly persistent in the environment and bio-accumulate in living organisms. Typical examples are chlorinated POPs such as organochlorine pesticides (OCPs), polychlorinated biphenyls (PCBs), or dioxins, brominated POPs such as polybrominated diphenyl ethers, or fluorinated POPs such as perfluoroalkyl and polyfluoroalkyl substances.

Although toxicity of high dose POPs is well-known, the recent concern is the possibility of adverse effects of low dose POPs, similar to the current environmental exposure levels. Among many diseases suspected to be linked to low dose POPs, type 2 diabetes (T2D) is the most convincing, given substantial evidence from both epidemiological and experimental studies.

Since 2010, we identified nine published review articles about human studies on POPs and T2D (1–9). Therefore, rather than adding one more ordinary review article on the same topic, this narrative review was written to bring up several provocative issues which are commonly missed by epidemiologists on POPs and T2D.

First, we briefly summarize the nine published review articles on POPs and T2D based on their own conclusions, rather than presenting our own quantitative analyses. Then we discuss (1) the importance of POPs as mixtures, (2) the role of POPs in inducing beta-cell dysfunction, (3) multi-dimensional interrelationships between POPs and adipose tissue, and (4) non-linear dose response relationships. Consideration of these aspects leads us to debate why currently prevailing actions (including regulations and suggestions for avoidance of POPs) focused on individual chemicals are not effective in protecting the public from POPs-related risk.

In this review, the focus is low dose environmental exposure, not occupational or accidental high dose exposure. Also, among various POP compounds, we deal with chlorinated POPs because they are ones which have shown the most consistent results in human studies. The evidence from brominated or fluorinated POPs is weak and inconsistent (9). Thus, unless we specify brominated or fluorinated, POPs means chlorinated POPs in this review.

## A brief summary of review articles on human studies on POPs and T2D

Table 1 summarizes conclusions from review articles about POPs and T2D which were published since 2010 (1–9). Sentences in the “Conclusion” column were extracted from abstract, highlights, and main findings without any modification. Some reviews focused on POPs while others covered a wide range of endocrine disrupting chemicals (EDCs; POPs were considered to be EDCs).

**Table 1:** Summary of review articles about epidemiological studies of persistent organic pollutants (POPs) and type 2 diabetes (T2D).

**Authors**	**Publication year**	**Verbatim conclusion from each review**
Taylor et al. (1)	2013	The overall evidence is sufficient for a positive association of some organochlorine POPs with type 2 diabetes.
Wu et al. (2)	2013	These findings support an association between POP exposure and the risk of T2D.
Lee et al. (3)	2014	The evidence as a whole suggests that, rather than a few individual POPs, background exposure to POP mixtures-including organochlorine pesticides and polychlorinated biphenyls-can increase T2D risk in humans.
Magliano et al. (4)	2014	In summary, while the overall evidence is strongly suggestive of an independent relationship between POPs and diabetes, some inconsistencies exist.
Ngwa et al. (5)	2015	Despite different levels of risk in prospective studies and inconsistent results, the causal effect of POPs on diabetes is supported by *in-vitro* and *in-vivo* experimental studies.
Jaacks et al. (6)	2015	The literature suggests a positive association between select POPs and diabetes.
Song et al. (7)	2016	Serum concentrations of persistent EDCs^*^ were significantly associated with T2D risk.
Evangelou et al. (8)	2016	Data suggest an association between organochlorine exposure and type 2 diabetes
Lind et al. (9)	2018	Evidence is accumulating that EDCs^*^ might be involved in diabetes development. Best evidence exists for p,p'-DDE.

* *EDCs (endocrine disrupting chemicals), POPs are classified as EDCs*.

Although they include systemic reviews, meta-analyses, and narrative reviews, all reviews concluded that there is potentially a role of POPs in the development of T2D despite some inconsistencies and research gaps. However, there are several critical issues which have not been properly considered in most original articles and reviews about POPs and T2D. As they can substantially influence the establishment of causality, the discussion of these issues can be helpful to understand the findings from published articles and plan future epidemiological studies.

## Critical issues for human studies of POPs and T2D

### POPS as a surrogate marker of lipophilic chemical mixtures

Most human studies about POPs and T2D have adopted individual chemical-based analyses. Namely, they measured serum concentrations of several or dozens of compounds belonging to POPs, evaluated individual associations between specific compounds and T2D, and interpreted their results focusing on those specific compounds which showed statistical significance. As a result, most reviews also followed the same strategy. In some reviews, only specific compounds such as hexachlorobenzene, p,p'-DDE, and PCBs, but not others, were considered to be linked to the risk of T2D (2, 9).

However, this approach should be reconsidered because recent human studies about POPs and T2D have been performed among general populations. It is well-known that there are substantial positive correlations among serum concentrations of various POP compounds (10, 11). Therefore, the epidemiologic findings on POPs should not be interpreted from the viewpoint of individual compounds which were directly measured and demonstrated statistical significance. The key feature of environmental exposure to POPs is the chronic exposure to the mixture of a variety of lipophilic chemicals at low dose even though absolute concentrations of individual compounds are variable. Focusing only on several compounds can largely distort the whole picture.

In most epidemiological studies, serum concentrations of POPs have been used as an exposure marker of POPs. Although concentrations of most synthetic chemicals measured in blood or urine are indicators of the current or recent exposure levels from the environment, serum concentrations of POPs are different. Humans are exposed to POPs through external exposure sources such as POPs-contaminated food. However, once POPs enter the body, they are primarily stored in adipose tissue and slowly released into the circulation to be eliminated over several years (12). Therefore, ultimately serum concentrations of POPs are largely determined by (1) the amount of POPs released from adipose tissue to circulation and (2) the amount of POPs eliminated from circulation.

In this situation, the findings about POPs in human studies should be interpreted beyond the compounds which are directly measured in each study. Measured POPs should be considered as surrogate markers of lipophilic chemical mixtures which include measured POPs, but also include unmeasured ones. This point has an important implication regarding how to approach the issue of POPs to protect the public. For example, even if we succeeded in completely eliminating specific POPs, this may not lead to the decrease of POPs-related disease because we have done nothing with other lipophilic chemicals which coexist with the eliminated POPs in the mixture. This aspect of POPs suggests that the most effective public health action to reduce harms from POPs should target lipophilic chemical mixtures.

### Role of POPs in inducing beta-cell dysfunction

T2D is increasingly recognized as a heterogeneous condition, ranging from predominantly insulin resistance with relative insulin deficiency to predominantly an insulin secretory defect with insulin resistance (13). Although most epidemiological studies about POPs have evaluated T2D as a whole, the role of POPs may differ depending on subtypes of T2D.

Obesity is the most important risk factor for insulin resistance-dominant T2D, however the role of adiposity is weak in T2D preceded predominantly by beta-cell dysfunction (14). Genetic predisposition has been considered a key determinant of beta-cell function (15), but the role of genes is still largely unknown despite many genome-wide association studies(16).

Environmental chemicals such as POPs can play a role in the development of beta-cell dysfunction (17). Although animal experimental studies have reported that the exposure to POPs can induce insulin resistance (18, 19), human studies evaluating both insulin resistance and insulin secretion have reported that serum concentrations of POPs were more strongly associated with decreased insulin secretion than with increased insulin resistance (20–23). An *in-vitro* cell study demonstrated that POPs can directly reduce insulin secretion at very low dose such as 1 pmol/L (23).

As decreased insulin secretion is a necessary step in developing both types of T2D, POPs can explain both types of T2D. However, the role of POPs may be more salient in beta-cell dysfunction-dominant T2D than insulin resistance-dominant T2D because the overproduction of insulin by pancreatic beta-cells during insulin resistance can mask the direct effect of POPs on beta-cell function (23). Also, POPs can explain why beta-cell dysfunction-dominant T2D is common in Asian and elderly people (24–26) who tend to have high serum concentrations of OCPs (27, 28).

### Multi-dimensional aspects of interrelationships between POPs and adipose tissue

POPs have been evaluated as a new risk factor for T2D, independent of traditional risk factors for T2D such as obesity or lack of physical activity. As a result, in epidemiological studies of POPs and T2D, obesity has been considered as a confounder. However, obesity cannot be a simple confounder in the relationship between POPs and T2D. The role of POPs should be comprehensively evaluated and interpreted, considering possibly interactive roles with obesity, due to their innate interrelationship.

Under the current paradigm, obesity is a key risk factor for many insulin resistance-related diseases such as T2D. Several mechanisms explain how obesity can increase these diseases. First, obesity can induce chronic inflammation of adipose tissue and release pro-inflammatory cytokines (29). Second, obesity increases the release of free fatty acid to circulation and promotes fat deposits in ectopic sites such as liver, muscle, and pancreas (30).

**Figure 1:**
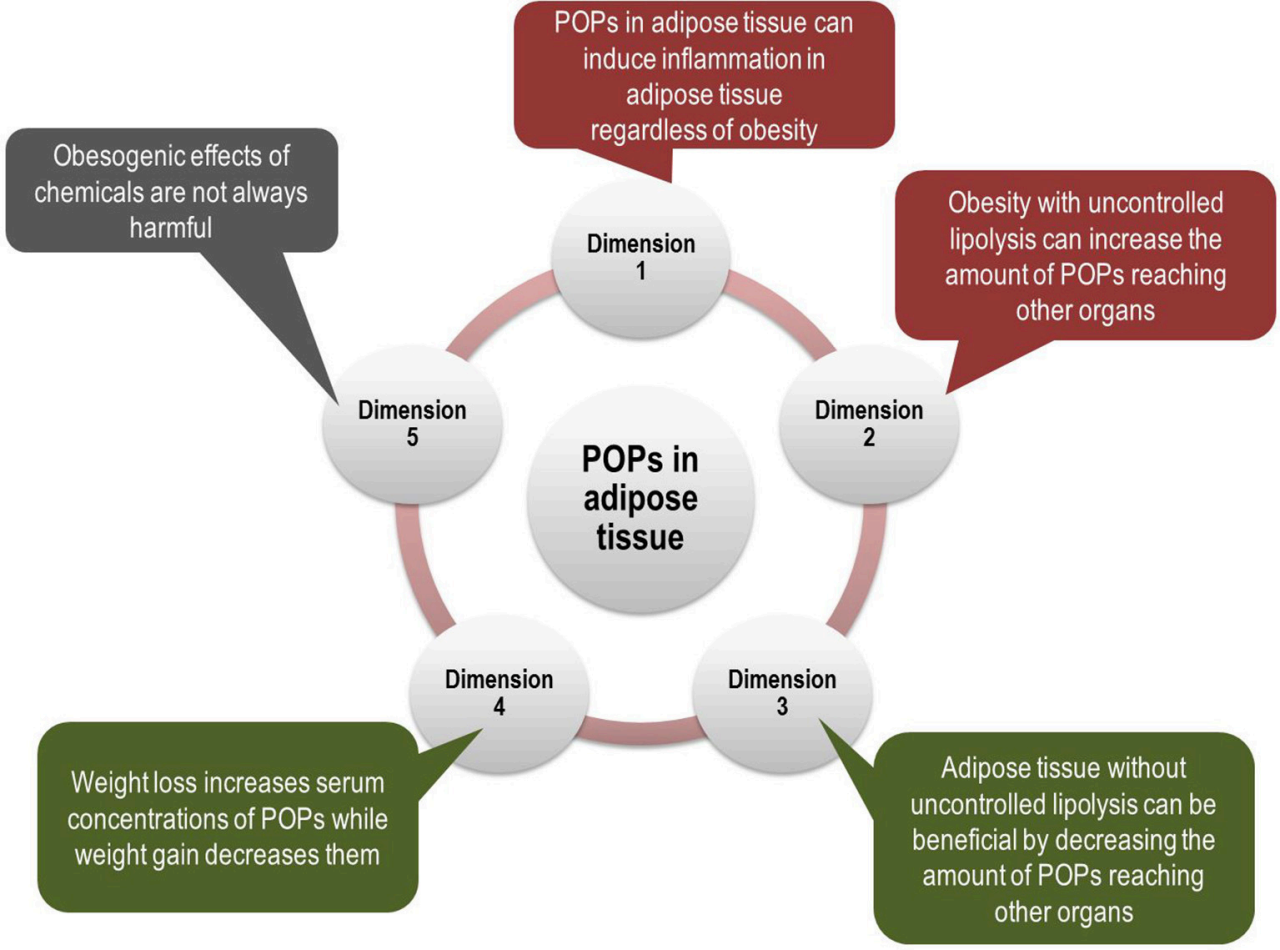
Multi-dimensional aspects of interrelationships between persistent organic pollutants (POPs) and adipose tissue. Even though POPs have been evaluated as a new risk factor for type 2 diabetes (T2D), independent of obesity, the role of POPs should be evaluated together with obesity due to their innate interrelationship. POPs are involved in key mechanisms linking obesity and T2D such as pro-inflammatory adipose tissue and ectopic fat accumulation (Dimensions 1 and 2). In addition, POPs can explain puzzling findings about obesity, which suggest beneficial effects of adipose tissue (Dimensions 3 and 4). Finally, obesogenic effects of chemicals may not always be harmful (Dimension 5). All these issues are critical to understand the role of POPs in the development of T2D.

However, POPs may participate in all these mechanisms, explained in detail below. Also, POPs can explain some puzzling findings on obesity which cannot be explained by the current paradigm of obesity. We present five different possible dimensions linking POPs and adipose tissue (Figure 1). Among them, dimensions 1 and 2 show that POPs can explain traditional obesity-related harmful effects while dimensions 3 and 4 show that POPs can explain puzzling findings on obesity. Dimension 5 casts a question on the current prevailing viewpoint which directly links obesogen-inducing chemicals to diabetes-inducing chemicals. All these issues are critical to understand the role of POPs in humans in a more comprehensive way and also to contrive effective ways to protect the public from POPs.

#### Dimension 1: POPs in adipose tissue can induce inflammation in adipose tissue

Adipose tissue is not only a reservoir of chronic internal POPs exposure, but also a possible tissue pathologic target of POPs (31). *In-vivo* and *in-vitro* experimental studies reported that low dose POPs can induce pro-inflammatory change in adipose tissue (18, 19, 31–34). Importantly, POPs-induced inflammation is possible regardless of obesity (19).

A topic for future studies is the interesting speculation that obesity can exacerbate the inflammatory effects of POPs. For example, if POPs are released from lipid droplets of adipocytes to interstitial spaces through necrosis of the hypertrophic adipocytes which are common among obese persons (35), the existence of poorly degradable foreign bodies such as POPs can trigger an adipose tissue immune response.

#### Dimension 2: obesity with uncontrolled lipolysis can increase the amount of POPs reaching other organs

At normal physiological conditions, lipolysis rates of adipose tissue are tightly regulated through hormonal signals depending on caloric need (36). However, among obese persons, uncontrolled lipolysis is common and increased lipolysis accelerates the efflux of free fatty acids from adipose tissue to the circulation and ectopic fat accumulation (37). Insulin resistance can further enhance lipolysis in adipocytes (38).

Importantly, POPs are also released from adipocytes to circulation during lipid mobilization (39) and POPs in circulation can easily reach other organs (40). Although on average there is a higher risk of uncontrolled lipolysis among obese persons than lean persons, even in non-obese persons, the release of POPs to circulation can increase if uncontrolled lipolysis exists. Toxicokinetics of POPs during lipolysis should be considered in all mechanisms related to lipolysis of adipose tissue.

#### Dimension 3: adipose tissue without uncontrolled lipolysis can be beneficial by decreasing the amount of POPs reaching other organs

In contrast to Dimension 2, as far as adipocyte function is physiologically healthy without uncontrolled lipolysis is concerned, greater adipose tissue can be more advantageous than less adipose tissue. The reason is that the storage of POPs in adipose tissue can reduce the amount of POPs reaching other organs (41) even though the storage of POPs in adipose tissue can have a negative side effect such as increasing half-lives of POPs in the long-term.

This aspect of adipose tissue may be helpful in explaining the obesity paradox (42): a better survival among overweight or obese patients or elderly despite a role of obesity as an important risk factor for many chronic diseases. The obesity paradox is also observed among patients with T2D, the exemplary obesity-related disease (43). Although several mechanisms such as body composition, cardiorespiratory fitness, or nutritional status have been suggested as possible explanations for the obesity paradox (42), POPs can provide a better explanation because the beneficial role of adipose tissue may be more important in patients or elderly than in healthy young persons. General features of patients with chronic diseases or elderly are the disturbance of homeostasis. In these conditions, the safe storage of pollutants in adipose tissue can be crucial. Supporting this speculation, there is a human study which showed that the beneficial role of adipose tissue may be increasing as serum concentration of POPs increases (44).

#### Dimension 4: weight loss increases serum concentrations of POPs while weight gain decreases them

In Dimension 2, we discussed that obesity can increase the release of POPs from adipose tissue to circulation through uncontrolled lipolysis. Ironically, weight loss also increases serum POPs concentrations (45, 46). On the other hand, weight gain can decrease them by sequestering POPs from the circulation into adipose tissue (40, 47). Therefore, possible effects due to dynamics of POPs during weight change are contrary to conventional expected effects of weight change from the viewpoint of fat mass. In that viewpoint, intentional weight loss is beneficial while weight gain is harmful.

Even though the improvement of metabolic risks with weight loss is well-known, the toxicokinetics of POPs during weight loss can negatively affect resting metabolic rate, thyroid hormone levels, and the oxidative potential of skeletal muscle (45, 48, 49). Also, it can be helpful to explain why the intensive lifestyle intervention focusing on weight loss among overweight or obese patients with T2D failed to show a decrease of the development of cardiovascular diseases, compared to the usual management group (50). Repeated experience of weight loss and gain would be the most serious concern due to tissue redistribution of POPs between adipose tissue and other organs (40).

However, the different release pattern of POPs between Dimension 2 and Dimension 4 warrants further discussion. The release of POPs among obese persons through uncontrolled lipolysis is chronic and subtle while the release of POPs during intentional weight loss is temporary and large. Compared to the release of a large amount of POPs for a short period time, the release of a small amount of POPs for a long period can be more harmful because POPs-related health effects do not seem to increase linearly with increasing dose of POPs, as discussed below. In addition, exercise and changes in diet habit which are commonly accompanied with intentional weight loss can increase the elimination of POPs (51) and mitigate possible harms of POPs (52).

Importantly, even though it is intentional, weight loss among the elderly can be problematic. Elderly people can release more POPs into circulation than younger people because of more contamination of adipose tissue with POPs (27), but the capability of metabolizing and excreting xenobiotics is decreasing with aging (53). Therefore, POPs in circulation in the elderly can more easily reach other lipid-rich organs such as the brain. Recently, the dynamics of POPs has been suggested as a key factor to explain a high risk of dementia among patients with T2D as well as puzzling findings on obesity and dementia (54).

#### Dimension 5: obesogenic effects of chemicals may not always be harmful

Recently, obesity-inducing effects of environmental chemicals including POPs have gained attention from researchers (55). Obesogens typically act at environmentally relevant doses during critical windows of prenatal or postnatal development to promote obesity later in life and these effects can be transmitted to their descendants (56). Possible mechanisms include increasing number of fat cells, increasing size of fat cells, and modulating hormones affecting appetite, satiety, and energy metabolism (57).

The current prevailing viewpoint on obesogens is that they can also act as diabetogens because they contribute to the development of obesity (58). However, the role of adipose tissue to protect other organs from POPs (Dimension 3) should be incorporated in interpreting the implication of obesogens. Among two mechanisms of the adipose tissue expansion, hypertrophy-dominant obesity (increase in cell size) is harmful due to pro-inflammatory cytokine release and impaired insulin sensitivity, but hyperplasia-dominant obesity (increase in cell number) is known to be beneficial (59). One of the key mechanisms by which obesogens promote adiposity is by increasing adipogenesis (56). Therefore, as far as they can provide healthy adipose tissue, their adipogenesis-promoting effect is not in itself necessarily harmful.

Although the obesogenic effect of DDT and their metabolites have been reported in human, *in-vivo*, and *in-vitro* studies (60), some POPs can suppress adipogenesis (33, 61). From the viewpoint of Dimension 3, these chemicals can be more harmful than obesogens if they diminish the safe storage site for lipophilic chemicals. The reality is much more complicated. For example, the same chemicals can increase adipogenesis at low dose while they can inhibit adipocyte differentiation at high dose (32). Also, there are synergic or antagonistic effects on adipogenesis based on studies of only two chemicals (33). Therefore, it would be almost impossible to predict what will be the net effect of chemical mixtures on the development of obesity.

As we discussed in Dimension 1, adipocytes were observed to be in a pro-inflammatory state regardless of whether POPs induced adipogenesis or reduced adipogenesis (32, 33). Therefore, rather than linking POPs to obesogens, investigation of direct harmful effects of POPs on adipose tissue and other organs would be more worthwhile in future research.

### Non-linearity

Body burden of OCPs and PCBs in current general populations are much lower than they were in the era when these chemicals were actively used. Then, why do current low dose POPs reveal significant associations in human studies?

Interestingly, risks of many common environmental chemicals do not increase linearly within the range of environmental low dose exposure (62–64). POPs have also demonstrated the possibility of non-linearity with T2D (3). Within the low dose range, the risk of disease increases linearly within the relatively low dose range, but it does not further increase as dose increases. It can slow down, flatten, or even decrease with increasing doses. This feature is different from the linear dose-response relationship which is commonly observed in the range of high dose toxicity.

This non-linearity has been considered to be biologically implausible and was criticized as artifactual, arising from confounding or bias (65, 66). However, the non-linearity is plausible through several mechanisms. First, if chemicals are harmful through endocrine disruption, they can show non-linearity (67). Second, the activation of stress responses with certain levels of chemicals, called hormesis, can be another mechanism to explain non-linearity (68). The traditional concept of hormesis does not consider possible harmful effects at sub-hormetic very low dose, but only contrasts “beneficial low dose zone” vs. “toxic high dose zone” (69). Within the environmental exposure range, the non-linearity can be the result of the combination of a “sub-hormetic harmful zone” and a “hormetic beneficial zone.”

Epidemiologists should understand how non-linearity can affect human studies. Historically, populations with high dose exposure to chemicals have been considered as optimal for investigation of the association between chemical exposure and outcomes. Under non-linearity, however, the association between any exposure and disease should be studied among populations with low dose exposure because the exposure range would correspond to the low dose, linear part of the dose-response relationship. In populations with relatively high dose exposure, the observed result may well be a null association because the exposure range would be in the flattened, higher dose part of the dose-response relationship. This kind of non-linearity can be one reason why current general populations with low body burden of OCPs and PCBs show clear results.

Under non-linearity, conventional quantitative methods of reviews such as meta-analyses or pooled-analyses can be problematic because they assume linearity when summarizing results such as relative risks or odds ratios from individual studies which have been performed in various populations with different levels of POPs.

## POPs as mitochondrial toxin, not conventional EDCs, can better explain human findings on POPs and T2D

POPs are well-known EDCs and T2D is an endocrine disease. Therefore, it is common to consider that POPs would be linked to T2D as EDCs, as seen in the titles of some review articles (7, 9). However, it may be difficult to explain human findings about POPs and T2D by the endocrine disrupting properties of POPs.

Several EDCs acting on the same pathway, such as estrogenic chemicals, have gained a lot of attention from researchers due to strong synergic effects (70). However, the unpredictable antagonistic effects of EDCs acting on different pathways are largely ignored (71–74). For example, the mixture of even two different EDCs (estrogenic and androgenic), did not produce predictable mixture effects. POPs are the mixture of diverse EDCs acting as either agonists or antagonists on different hormone receptors which can engage in crosstalk with each other. Therefore, it would be impossible to reliably evaluate the net effect of POPs acting as EDCs in humans.

Considering the complexity of EDC mixtures, other mechanisms might be more plausible as an explanation for the consistent findings about POPs relating to T2D in human studies. In fact, many environmental chemicals are known as mitochondrial toxins (75). Evidence is accumulating that low dose POPs can induce mitochondrial dysfunction and/or reduce oxidative phosphorylation capacities (18, 76–78). Although high dose POPs are well-known direct mitochondrial toxins (79), recent studies highlight functional impairment of mitochondria by low dose POPs. In fact, endocrine disruption can occur as a result of mitochondrial dysfunction because mitochondria are essential sites for steroid hormones biosynthesis in the steroidogenic cells (80).

Mitochondrial dysfunction is currently linked to many common age-related chronic diseases including T2D (81). However, the functional impairment of mitochondria can be reversed by restoration of their function. In the section “Non-linearity,” we discussed hormetic stress responses as a mechanism to explain the flattened part of the dose-response curve in the higher part of the low dose range. The activation of mitochondrial function is a typical example of hormetic stress response (82). Therefore, the non-linearity observed in the association between POPs and T2D can be explained by dynamics of mitochondrial function.

Particularly, it is important to note that there are health behaviors which can improve mitochondrial function (83). They include exercise, calorie restriction, and intake of phytochemicals in plant food (84–86). At present, regardless of POPs, these health behaviors are known as beneficial to decrease the risk of T2D and glycemic control among T2D patients. However, POPs may be a piece of the puzzle which has been missed in the relation between healthy behaviors and T2D.

## Perspective on future studies of POPs

At present, researchers tend to think that even a small increased risk of T2D among persons due to POPs will have huge public health impact because of the ubiquitous presence of POPs and the high prevalence of T2D. However, as issues related to POPs cannot be separated from obesity issues, as we discussed above, a more comprehensive viewpoint about POPs is needed.

As the majority of published human studies about POPs and T2D are still cross-sectional, more prospective studies on POPs and T2D would be desirable. The role of POPs can be better studied among populations in which beta-cell dysfunction-dominant T2D is common, such as Asians or the elderly. In particular, the relationship between POPs and obesity on the development of T2D should be thoroughly investigated. However, the value of long-term follow-up may not be as good as epidemiologists generally believe for reasons discussed below.

First, as the dynamics of adipose tissue continuously affect serum concentration of POPs, the baseline POP value may become less representative of POPs exposure as the follow-up period gets longer. In modern society, many persons repeatedly experience weight gain and weight loss. This experience would be more frequent among obese persons, a high risk group for T2D. Therefore, although POPs are an example of synthetic chemicals with reliable exposure assessment due to their long half-lives (87), the value of baseline serum concentrations of POP decreases as follow-up period gets longer.

Second, in the case of exposure variables such as POPs which are expensive to measure, a nested case control study is generally regarded as the best design for human studies. However, as the value of measurement of POPs at baseline is decreased when the follow-up period increases for the reason we discussed above, the value of a nested case control study is also limited. Additionally, serum stored in periods when POPs were actively used would not be advantageous due to their high serum concentrations among study subjects. Under non-linearity, populations with low dose exposure are better to explore the relationship between exposure and disease than are populations with high dose exposure.

Third, the effects of POPs on the development of T2D may be dynamic. Unlike high dose toxicity with irreversible damage, functional impairment due to low dose exposure can be reversible. For example, as we discussed above, healthy lifestyles can reverse harmful effects of POPs through the improvement of mitochondrial function. Therefore, the interaction between healthy behaviors and POPs can dynamically influence the development of T2D.

All these issues suggest that a prospective study or a nested case control study with only baseline information may fail to uncover a role of POPs in the development of T2D. This problem becomes more serious as the follow-up period gets longer. Considering the necessity of repeated measurements of POPs, obesity, and other relevant variables, epidemiologists need to plan a reasonable follow-up period. The development of cheap and quick bioassay methods to assess POPs in bio-specimens would be useful for large-scale human studies with repeated measurements.

Considering the difficulties in human studies, laboratory studies mimicking human exposure would be crucial. For this purpose, animal models using lipolysis of adipose tissue such as hypertrophic adipocytes with uncontrolled lipolysis or repeated weight cycling can be useful. Mitochondrial dysfunction by the environmentally-relevant doses of POPs and the possibility of their restoration by the activation of mitohormesis would be another important study topic in the field of laboratory research.

## Conclusion

If POPs are a new risk factor for T2D, what can we do? POPs are different from traditional risk factors such as cigarette smoking which can be avoided through education, policy, etc. In modern society, complete avoidance of exposure to POPs is not possible due to the wide contamination of the food chain. Also, a large amount of POPs is already stored in our adipose tissue and they are continuously released to circulation. Furthermore, most problematic POPs such as OCPs or PCBs were already banned in most countries several decades ago.

Therefore, how to manage POPs in adipose tissue and circulation is the foremost issue in humans. Routine application of health behaviors such as exercise, calorie restriction, and high intake of phytochemicals have been suggested as practical ways to increase the continuous elimination of POPs from human bodies or mitigate harmful effects of POPs at the cellular level based on physiology of metabolism of xenobiotics and mitochondrial function (51, 52). Even though their effects need to be evaluated in randomized controlled trials, short-term clinical trials may fail to discover solid evidence due to the dynamic nature of serum concentrations of POPs. However, as these behaviors are currently well-accepted as healthy even without any consideration on POPs, they can be safely recommended to the public. Besides health behaviors, future research focusing on the development of more effective methods of eliminating POPs is needed.

## Author contributions

Y-ML wrote the first draft of the manuscript. DJ contributed to the revision of draft. D-HL devised the main concept of manuscript and contributed to the revision of draft. All read and approved the submitted version.

### Conflict of interest statement

The authors declare that the research was conducted in the absence of any commercial or financial relationships that could be construed as a potential conflict of interest.
